# Tumor-associated CD8^+^T cell tolerance induced by erythroid progenitor cells

**DOI:** 10.3389/fimmu.2024.1381919

**Published:** 2024-05-10

**Authors:** Xue Fan, Han Peng, Xuesong Wang, Yixin Sun, Yan Dong, Jie Zhou, Jianfang Chen, Shuo Huang

**Affiliations:** ^1^Department of Oncology and Southwest Cancer Centre, Southwest Hospital, Third Military Medical University (Army Medical University), Chongqing, China; ^2^Radiation Treatment Centre, Southwest Hospital, Third Military Medical University (Army Medical University), Chongqing, China; ^3^Endocrinology/Osteoporosis Department, West China School of Public Health and West China Forth Hospital, Sichuan University, Chengdu, China

**Keywords:** erythroid progenitor cell, CD45, peroxynitrite, immunotherapy, CD8^+^T cell

## Abstract

**Introduction:**

CD8^+^T cell tolerance plays an important role in tumor escape. Recent studies have shown that CD45^+^ erythroid progenitor cells (CD45^+^EPCs) generated through splenic extramedullary erythropoiesis suppress tumor immunity. However, the mechanism underlying how CD45^+^EPCs mediate CD8^+^T cell tolerance remains incompletely understood and requires further research.

**Methods:**

In this study, the antigen-processing abilities of CD45^+^EPCs was verified through both in vitro and in vivo experiments. We have used the method of co-culture in vitro and adoptive transfer experiments in vivo to explore the effects of CD45^+^EPCs on CD8^+^T cell tolerance. RNA-sequencing analysis and blocking experiments were used to evaluate the role of ROS in the CD45^+^EPC mediated tolerance of CD8^+^T cells. Finally, we incorporated uric acid into the adoptive transfer experiments to rescue the CD45^+^EPC mediated tumor-promoting effect.

**Results and discussion:**

We found that CD45^+^EPCs take up soluble proteins, present antigenic epitopes on their surface, and induce antigen-specific CD8^+^T cell anergy. In addition, we found that CD45^+^EPC directly nitrates tyrosine within the TCR/CD8 complex via the production of reactive oxygen species and peroxynitrite, preventing CD8^+^ T cells from responding to their specific peptide antigens. Furthermore, uric acid treatment effectively abolished the immunosuppressive effects of CD45^+^EPCs during CD8^+^T cell adoptive transfer, thereby enhancing the anti-tumor efficacy. These results demonstrated that CD8^+^T cell tolerance in tumor-bearing mice is induced by CD45^+^EPCs. The results of this study have direct implications for tumor immunotherapy.

## 1 Introduction

Tumor immunosuppression is induced by CD8^+^T cell tolerance, which plays a major role in tumor escape (1–3), as well as by the secretion of various immunosuppressive cytokines by tumor cells into their microenvironment, limiting the success of immunotherapy (3). Previous studies have shown that antigen-presenting cells (APCs) are responsible for inducing tumor-induced T-cell tolerance. APCs, including myeloid-derived suppressor cells (MDSCs) (4), immature dendritic cells (iDCs) (5) and tumor-associated macrophages (6), are generated in the bone marrow and accumulate in the lymph nodes, spleen, and tumor tissues (7). In addition, APCs in the tumor microenvironment not only secrete immunosuppressive factors but also pick up and process soluble proteins, thereby inducing antigen-specific tolerance in CD8^+^T cells (8).

CD45^+^ erythroid progenitor cells (CD45^+^EPCs) are abundant in the spleen of neonatal mice and the umbilical cord blood of humans (9, 10). Interestingly, recent studies have shown that CD45^+^EPCs also exist in tumor-bearing mice and patients with cancer and contribute to immunosuppression (11–13). CD45^+^EPCs accumulate in the bone marrow, liver, blood, and tumor tissues of various tumor animal models and induce immunosuppressive effects by increasing reactive oxygen species (ROS) levels (12). Importantly, immunosuppressive genes have been found in CD45^+^EPCs and MDSCs (12). In addition, CD45^+^EPCs lose their erythroid development potential and switch to the myeloid lineage (MDSC). These findings led us to speculate that CD45^+^EPCs have similar characteristics to those of APCs, which can process antigens *in vivo* and induce antigen-specific tolerance in CD8^+^T cells.

The investigation of CD8^+^ T-cell tolerance in tumor-bearing hosts is limited by the nature of experimental models because the persistent effect of tumor-derived factors makes it difficult to investigate the role of defined cell populations. Therefore, we used a mouse model with the adoptive transfer of different cell types isolated from MC38 tumor-bearing mice into tumor-free recipients. In the current study, we demonstrated that CD45^+^EPCs from tumor-bearing mice can pick up soluble proteins, process them, and induce antigen-specific tolerance in CD8^+^ T cells. Mechanistically, we found that ROS and peroxynitrite generated by CD45^+^EPCs induce the nitration of tyrosine residues in TCR and CD8 molecules, inducing antigen-specific non-responsiveness of CD8^+^T cells. Furthermore, we found that uric acid (UA), which neutralizes peroxynitrite, effectively abolishes the immunosuppressive effects of CD45^+^EPCs during CD8^+^T cell adoptive transfer. Indeed, CD8^+^T cell adoptive immunotherapy combined with UA treatment achieved better anti-tumor efficacy than that of monotherapy. Overall, the present study reveals the immunosuppressive mechanism of CD45^+^EPC, which may have direct implications for tumor immunotherapy.

## 2 Materials and methods

### 2.1 Mice and reagents

Female C57BL/6, BALB/c mice(6-8 weeks of age) were purchased from the Chinese Academy of Medical Sciences (Beijing, China). OT-1 TCR-transgenic mice, Pmel transgenic mice, and CD45.1^+^ congenic mice were purchased from Jackson Laboratory. The mice were housed and maintained in laminar flow cabinets under specific, pathogen-free conditions. The mice were cared for and used in accordance with army medical university ethical guidelines. To establish tumor models, C57BL/6 mice were injected s.c. with MC38-OVA or B16 cells.

OVA-derived peptide(H-2Kb, SIINFEKL), control H-2Kb RAHYNIVTF peptides, and gp100 peptide were purchased from Sigma-Aldrich. All Abs used for flow cytometry were obtained from Biolegend. ROS inhibitor (apocynin) was purchased from MCE.

### 2.2 Cell lines and cell culture

B16 cells were obtained from the American Type Culture Collection (ATCC). MC38-OVA cells were generously provided by yue zhang. Cancer cells were cultured in DMEM/H(HyClone) supplemented with 10%FBS(Gibico) and 100 U/ml penicillin/streptomycin. CD8^+^T lymphocytes were isolated from spleens of wild type mice using T-cell enrichment columns (Stem Cell). To activate spleen or CD8^+^T cells, the cells were seeded in 96-well plate with OVA-derived peptide, control H-2Kb RAHYNIVTF peptides, or gp100 peptide. For T cell suppression assays, CD8^+^ T cells were labeled with CFSE and cultured in RPMI 1640 supplemented with 10% fetal bovine serum and 100 U/ml penicillin/streptomycin. And T cells were cocultured at 2:1 ratios with spleen-derived CD45^+^Ter119^+^CD71^+^cells (CD45^+^EPC) in 96-well flat-bottom plates. Seventy-two hours later, cells were analyzed by flow cytometry. In some coculture experiments, 10ug/ml OVA/H2Kb or gp100 peptides was seeded for the cell activation, and CD45^+^ EPCs were treated for 30 min before and during coculture with 300 mM apocynin to inhibit NADPH oxidase.

### 2.3 Adoptive transfer tumor experiment

In the experiment examining the specific immune tolerance of CD8^+^ T cells mediated by CD45^+^EPCs, CD45.2 naive mice recipients of OT-1 T cells(2x10^6^) were immunized with OVA or gp100. CD45^+^EPC cells were isolated from spleens of MC38 tumor-bearing mice, pulsed with specific or control peptides, and injected i.v. (2 x 10^6^) 8 days after immunization. Cells from splenocytes were re-stimulated with specific or control peptides and analyzed. In the CD45^+^EPC tumor-promoting experiment, MC38-OVA (1 × 10^6^) and B16 (2 × 10^5^) were implanted subcutaneously into female C57BL/6 mice. 5days later(Day-5), OT1 or PMEL CD8^+^T cells (2 x 10^6^) were intravenously transferred after tumor cell inoculation, and 2 days later, mice were immunized with OVA or gp100(100ug). The mice were then intravenously injected with specific peptide-pulsed CD45^+^EPC (2x10^6^) isolated from the spleens of tumor-bearing mice at Day2, 4, 6. In some experiments, UA treatment(20 mg/100 μl PBS) was started at day4 and continued for 7 days(Once a day for 7 consecutive days).

### 2.4 Cell isolation

Splenocytes were collected by mechanical disruption. Cell suspensions were passed through 70-μm cell strainers and then washed and resuspended in staining buffer. For the isolation of spleen CD45^+^Ter119^+^CD71^+^cells(CD45^+^EPC), cells were stained with anti-CD45 monoclonal antibody (mAb),anti-Ter119 (mAb), and anti-CD71 (both from Biolegend). Then, CD45^+^EPC were sorted using the BD FACSAsia II Special Order System. For spleen CD8^+^ T cell isolation, CD8^+^T lymphocytes were isolated from spleens of wild type mice using T-cell enrichment columns (Stem Cell). For all sorted samples, a purity of greater than 95% was achieved.

### 2.5 Flow cytometry

All antibodies are listed in **Supplementary Table 1**. Cells were blocked with rat IgG (10 μg/ml; Sigma) for at least 20 min on ice, washed with staining media [2%(vol/vol) HI, FBS in HBSS (BSS) without Ca^2+^ or Mg^2+^, denoted SM], and then stained with fluorescently-conjugated antibodies in SM for 30 min on ice. For the analysis of intracellular cytokines, CD8^+^T cells were re-stimulated with specific or control peptides. After 6 h, cell suspensions were stained using the Fixable Viability Dye (eBioscience) to remove dead cells and were surface stained for CD8^+^ using anti-mouse flow cytometry antibodies. For intracellular staining, cells were fixed and permeabilized in Fixation/permeabilization solution (BD Pharmingen) for 30 min. Then, cells were stained with anti-IFN-γ, Ki-67. All labeled cells were analyzed using a Beckman flow cytometry system.

### 2.6 Bioinformatic analysis

The transcriptomic expression profile was obtained from the GEO database using the accession numbers GSE106384 (mRNAs, tumor vs. wildtype). Differentially expressed genes (DEGs) between tumor and wildtype were identified using the “limma” R package with a threshold of |log2FC|>2 and adjusted p value<0.05. The R package “fgsea” was used to perform the GSEA with hallmark pathways from “msigdb” and the DEGs to investigate which hallmark pathways were significantly (p < 0.05) enriched in tumor group.

### 2.7 Statistical analysis

All statistical analyses were performed using GraphPad Prism 7.0 software. One- or two-tailed unpaired Student’s t tests and two-way ANOVA were used to compare two groups. Statistical significance was indicated as *p < 0.05, **p < 0.01, *** p < 0.001, and ****p < 0.0001. All experiments were independently repeated at least three times.

## 3 Results

### 3.1 CD45^+^EPCs can pick up and process tumor-associated antigens

**Figure 1:**
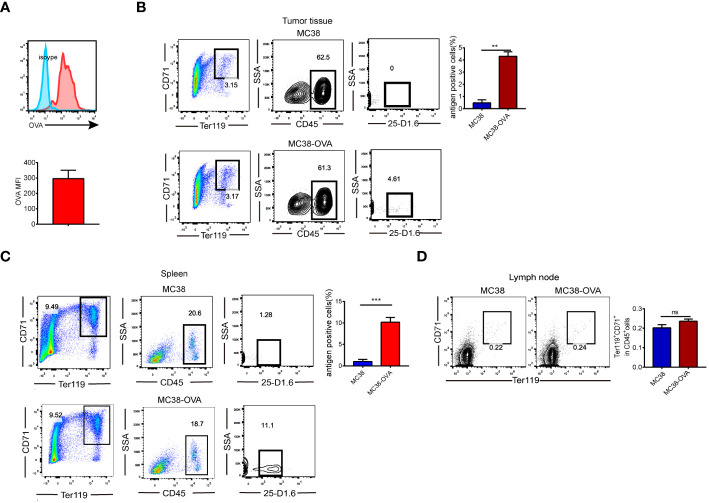
CD45^+^EPCs have the ability to pick up and process tumor-associated antigen. **(A)** CD45^+^EPCs take up soluble protein *in vivo*. Tumor-bearing mice were injected i.p. with 0.5 mg of OVA FITC. Percentage of OVA-positive cells was determined of CD45^+^EPCs. **(B, C)** MC38 or MC38-OVA tumor-bearing tumor model were established. When tumor size reached 2cm in diameter, splenocytes were analyzed using FACS. The percentage of CD45^+^EPC in tumor tissue and spleen expressing OVA-derived epitope was calculated by using anti-mouse H2-K^b^ bound to SIINFEKL antibody. Mean ± SEM is shown (n = 3). **(D)**, The proportion of CD45^+^EPC in the lymph nodes of tumor-bearing mice. Statistically-significant differences were measured by performing an unpaired Student’s t-test. **p < 0.01, ***p < 0.001. ns, means no significance.

We investigated whether CD45^+^EPCs could recognize soluble proteins. Ovalbumin (OVA) was intraperitoneally injected into MC38 tumor-bearing mice. Two hours later, increased OVA levels were detected in CD45^+^EPCs (**Figure 1A**), indicating that they could take up soluble OVA. Furthermore, we established a tumor model (MC38 and MC38-OVA) to demonstrate that CD45^+^EPCs could pick up and process tumor-associated antigens *in vivo*. When the tumor reached 2 cm in diameter, the cells from tumor tissue, spleen, and lymph node were analyzed using FACS. The percentage of CD45^+^EPC expressing the OVA-derived epitope was calculated using an anti-mouse H2-K^b^ antibody bound to the SIINFEKL antibody. Importantly, OVA-derived epitopes were detected in CD45^+^EPC in the tumor tissue and spleen from MC38-OVA tumor-bearing mice (**Figures 1B, C**). In the draining lymph nodes, there was almost no infiltration of CD45^+^EPCs in tumor-bearing mice (**Figure 1D**). These results indicate that CD45^+^EPC in the tumor tissue and spleen can capture and process tumor-associated antigens.

### 3.2 Antigen-specific nature of CD45^+^EPC-mediated CD8^+^T cell tolerance *in vitro*

**Figure 2:**
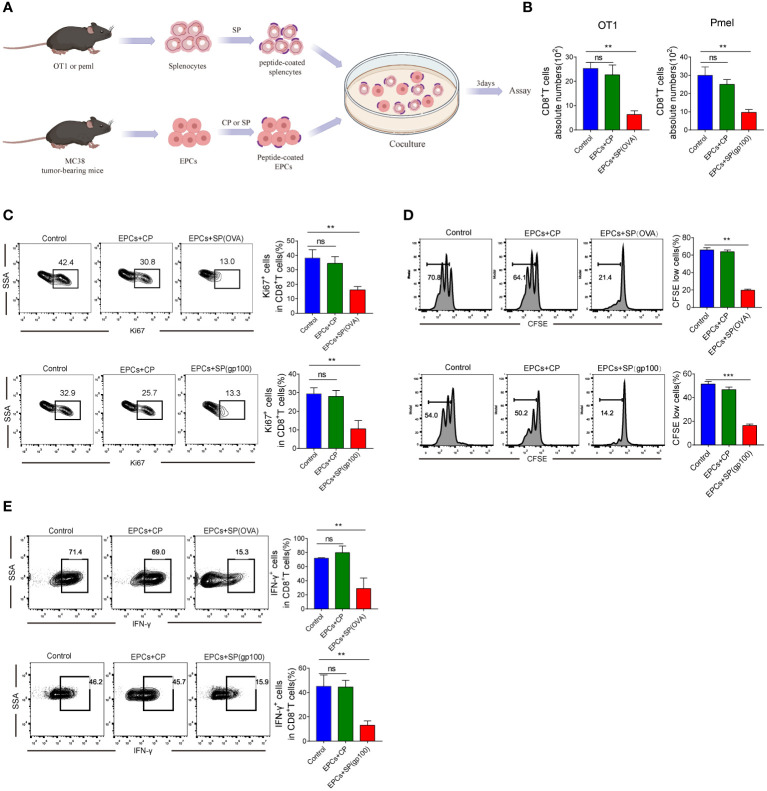
Antigen-specific nature of CD45^+^EPC-mediated CD8^+^T cell tolerance *in vitro*. **(A)** Schematic illustration of treatment schedule. **(B)** The absolute numbers of CD8^+^T cells. Mean ± SEM is shown (n = 3). **(C)** The proliferation percentage of CD8^+^T cells tested by Ki67 staining. Mean ± SEM is shown (n = 3). **(D)** The proliferation percentage of CD8^+^T cells tested by CFSE staining. Mean ± SEM is shown (n = 3). **(E)** IFN-γ expression of CD8^+^T cells was detected by IFN-γ staining. Mean ± SEM is shown (n = 3). Two-way ANOVA was performed to assess significance, **p < 0.01, ***p<0.001. ns, means no significance.

We investigated whether CD8^+^T cell activity was inhibited due to the presentation of specific peptides by CD45^+^EPC. CD45^+^EPC were pulsed with control or specific peptides (CP or SP) from MC38-ova or B16 tumor-bearing mice and co-cultured with splenocytes pulsed with specific peptides (**Figure 2A**). As expected, CD45^+^EPCs loaded with specific peptides reduced the absolute number of CD8^+^T cells and suppressed the expression of Ki67 (**Figures 2B, C**). Further, CD45^+^EPCs loaded with specific peptides effectively suppressed the proliferation of CD8^+^T cells (**Figure 2D**). No significant effect was observed in CD45^+^EPCs loaded with control peptides. The proportion of IFN-γ-expressing CD8^+^T cells decreased significantly when co-cultured with CD45^+^EPCs loaded with specific peptides (**Figure 2E**). These findings suggest that the administration of CD45^+^EPCs loaded with control peptides does not significantly affect the response of CD8^+^T cells, while CD45^+^EPCs loaded with specific peptides reduce CD8^+^T cell responses significantly.

### 3.3 Antigen-specific nature of CD45^+^EPC-mediated CD8^+^T cell tolerance *in vivo*

**Figure 3:**
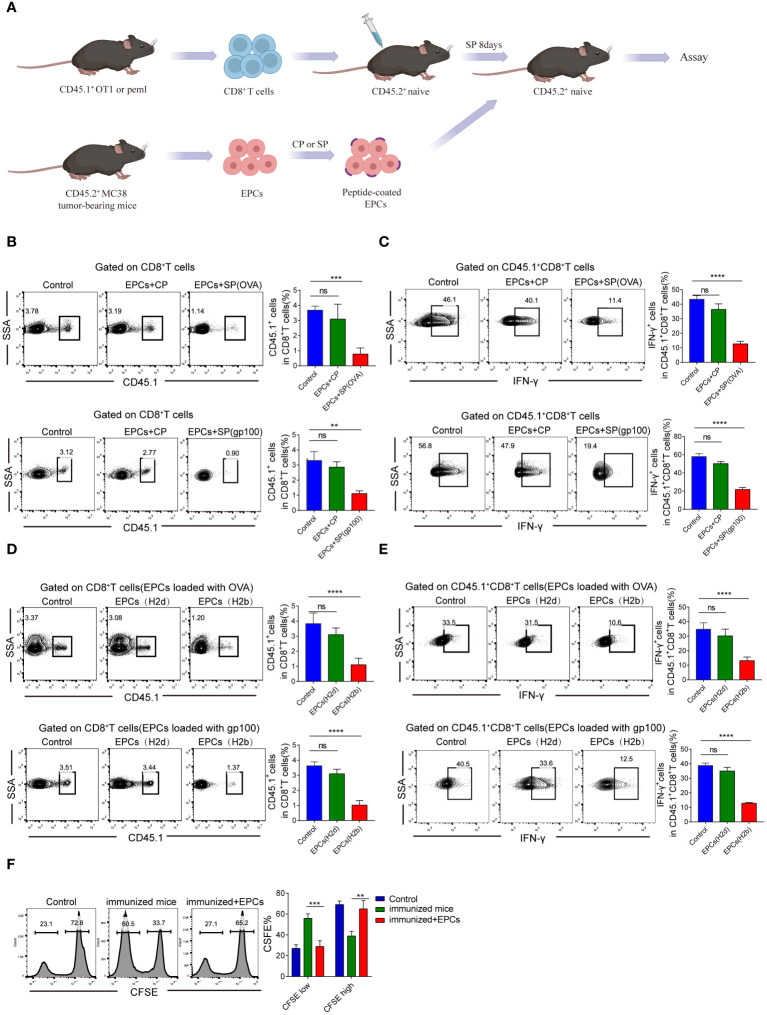
Antigen-specific nature of CD45^+^EPC-mediated CD8^+^T cell tolerance *in vivo*. **(A)** Schematic illustration of treatment schedule. **(B)** The percentage of OT1 or pmel CD45.1^+^CD8^+^T cells were analyzed by FACS using anti-CD45.1 and CD8 staining. Mean ± SEM is shown (n = 3). **(C)** IFN-γ expression of CD45.1^+^CD8^+^T cells was detected by IFN-γ staining. Mean ± SEM is shown (n = 3). **(D, E)** Pmel or OT-1 transgenic T cells (H2b) were transferred to F1 hybrid (C57BL/6 (H2b) BALB/c (H2d)) mice. Three days later, these mice have received CD45^+^EPC from either tumor-bearing C57BL/6 mice (H2b) or BALB/c mice (H2d), and at the same time were immunized with SIINFEKL peptide. After 10 days, the percentage of CD45.1^+^CD8^+^T cells and IFN-γ were tested by FACS. Mean ± SEM is shown (n = 3). **(F)** Cytotoxic activity *in vivo*. One group of splenocytes was pulsed for 2 h with 10 g/ml specific peptide (SIINFEKL), washed, and loaded with 5 uM CFSE. The other group was pulsed with the control peptide (RAHYNIVTF) and loaded with 1 uM CFSE. Cells were then mixed together at 3:1 ratio and injected i.v. into mice 10 days after immunization and CD45^+^EPC administration. Thirty-six hours later, spleens were collected and CFSE-positive cells were evaluated. Mean ± SEM is shown (n = 3). Two-way ANOVA was performed to assess significance, **p < 0.01. ***p < 0.001. ****p < 0.0001. ns, means no significance.

Next, we verified that CD45^+^EPCs mediated specific immunosuppressive effects *in vivo*. We established an *in vivo* model where the direct effect of CD45^+^EPCs on antigen-specific CD8^+^T cells could be evaluated *in vivo* (**Figure 3A**). In this model, OT-1 or PMEL T cells (CD45.1^+^) were transferred to naïve CD45.2^+^ congenic recipients. CD45^+^EPCs from MC38 tumor-bearing mice were transferred 8 days later, and the mice were immunized with specific peptides. Splenocytes were collected and re-stimulated with control or specific peptides after 24 h. The percentage of donor CD45.1^+^CD8^+^T cells in recipients of CD45^+^EPCs loaded with specific peptides decreased significantly (**Figure 3B**). CD45^+^EPCs loaded with specific peptides also inhibited the acquisition of effector phenotype by CD45.1^+^CD8^+^ T cells, which is evident from the downregulation of IFN-γ expression (**Figure 3C**). There are several potential explanations for down-regulation of CD8^+^T-cell function (e.g., anergy, exhaustion, or cell death), and further studies are needed to confirm the cause. We observed that CD45^+^EPCs loaded with specific peptides effectively inhibit the expression of CD44, TNF-α, and IL-2 on CD8^+^T cells (**Supplementary Figures 1A–C**). PD-1 and Tim3 are not expressed on CD8^+^T cells in all groups. (**Supplementary Figures 1D, E**). Based on these findings, we speculate that CD45^+^ EPCs down-regulate CD8^+^T cell function by inducing anergy. Moreover, we established a BALB/c × C57BL/6 mouse model to be used as a recipient of OT-1 or PMEL T cells. CD45^+^EPCs from MC38 tumor-bearing C57BL/6 or CT26 tumor-bearing BALB/c mice were used in these experiments. Mice immunized with specific peptides received CD45^+^EPC at the time of immunization and were evaluated 10 days later. The results showed that CD45^+^EPCs from BALB/c mice (H2^d^) did not suppress the response of OT-1 CD8^+^ T cells (H2^b^) to the H2K^b^-matching peptide, indicating that CD8^+^T cell response inhibition was MHC-I restricted. As expected, the CD8^+^ T cell response to the specific peptide was not inhibited in immunized mice after the administration of CD45^+^EPCs isolated from CT26 tumor-bearing BALB/c mice (**Figures 3D, E**). In contrast, the administration of CD45^+^EPCs loaded with specific peptides from MC38 tumor-bearing C57BL/6 mice decreased the percentage of CD8^+^T cells and the expression of effector molecules significantly (**Figures 3D, E**). We further investigated whether CD45^+^EPCs loaded with specific peptides affected the killing capacity of CD8^+^T cells. Splenocytes from C57BL/6 mice loaded with CFSE were used as targets. Splenocytes pulsed with the specific and control peptides were loaded with CFSE at concentrations of 5 and 1 µM. The two target cell lines were then transferred to C57BL/6 mice after immunization and CD45^+^EPC transfer. We found that the percentage of splenocytes pulsed with specific peptides was significantly reduced in immunized mice that did not receive CD45^+^EPCs but remained almost unchanged in those that did (**Figure 3F**). Taken together, these results demonstrate that CD45^+^EPCs induced MHC-I-restricted antigen-specific tolerance of CD8^+^T cells and abrogated CD8^+^T cell activity.

### 3.4 CD45^+^EPCs reduce the efficacy of CD8^+^T cell adoptive immunotherapy

**Figure 4:**
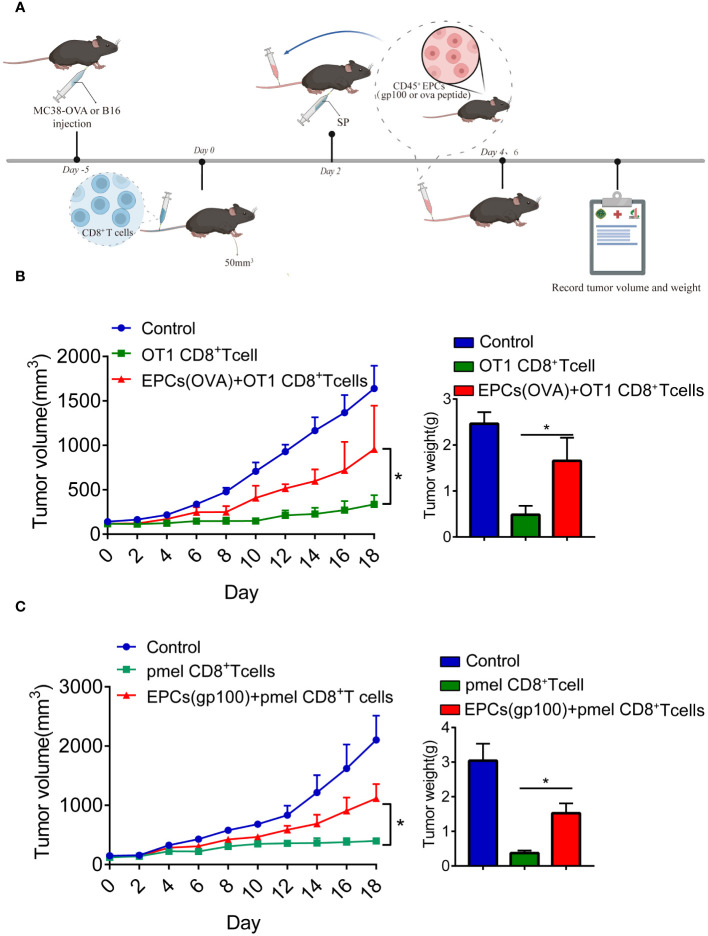
CD45^+^EPCs reduce the efficacy of CD8^+^T cell adoptive immunotherapy. **(A)** Schematic illustration of treatment schedule. MC38-OVA or B16 tumor-bearing mice received SIINFEKL peptide or gp100 peptide on the indicated days. CD8^+^T cells and CD45^+^EPC were transferred into mice on the indicated days. Results represent the assessment of tumor growth differences at the last time point. **(B, C)** Tumor growth analysis in tumor-bearing mice (n = 6). Two-way ANOVA was performed to assess significance,*p < 0.05.

Previous studies have reported that CD45^+^EPCs accelerate tumorigenesis (12, 13). Accordingly, we investigated the role of CD45^+^EPCs in antigen-specific CD8^+^T cell adoptive immunotherapy. MC38-OVA or B16F10 tumor cells were subcutaneously transplanted into C57BL/6 mice. On day 0, OT1 or PMEL CD8^+^T cells (2 × 10^6^) were intravenously transferred after tumor cell inoculation. After 2 days, mice were immunized with OVA or gp100 (**Figure 4A**). The mice were then intravenously injected with specific peptide-pulsed CD45^+^EPCs isolated from the spleens of tumor-bearing mice. We found that tumor growth was not restored in mice injected with CD45^+^EPCs and CD8^+^T cells (**Figures 4B, C**). These results suggest that CD45^+^EPCs reduce the efficacy of antigen-specific CD8^+^T cell adoptive immunotherapy.

### 3.5 Mechanism underlying CD45^+^EPC-induced CD8^+^T cell tolerance

**Figure 5:**
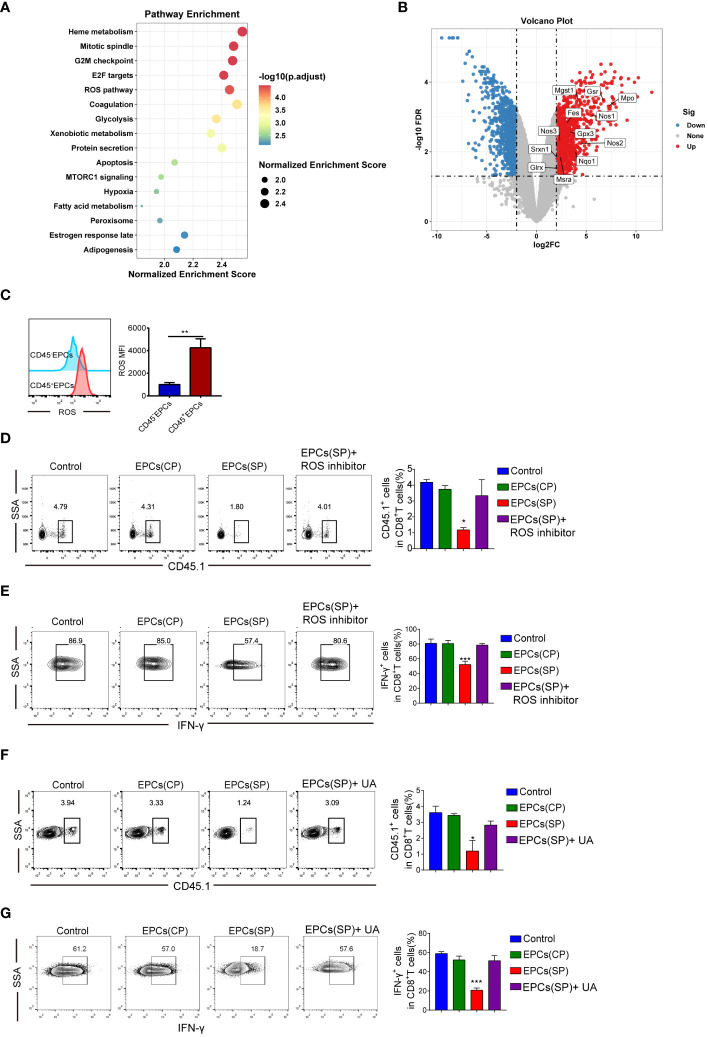
Mechanism of CD45^+^EPCs induced CD8^+^T cell tolerance. **(A)** Pathway enrichment analysis was performed using Gene Set Enrichment Analysis(GSEA). Significantly enriched items in CD45^+^EPCs derived from tumor-bearing mice compared with CD45^-^EPCs are shown with enrichment scores. **(B)** volcano plot of CD45^+^EPCs and CD45^-^EPCs from tumor-bearing mice. **(C)** ROS expression in CD45^+^EPCs and CD45^-^EPCs was detected by FACS. Mean ± SEM are shown (n = 3). **(D, E)** Adoptive transfer and immunization was performed in **Figure 2**. The percentage of CD45.1^+^CD8^+^T cells was analyzed by FACS using anti-CD45.1 and CD8 staining upon ROS inhibitor or UA treatment. Mean ± SEM are shown (n = 3). **(F, G)** IFN-γ expression of CD45.1^+^CD8^+^T cells was detected by IFN-γ staining upon ROS inhibitor or UA treatment. Mean ± SEM is shown (n = e). Two-way ANOVA was performed to assess significance, *p < 0.05, **p < 0.01, ***p < 0.001.

To study the molecular mechanisms underlying immunosuppression by CD45^+^EPCs, GSE106384 dataset was collected, In this dataset, RNA-seq was performed using CD45^+^EPCs and CD45^-^EPCs from the spleens of tumor-bearing mice and wild-type mice. RNA-seq data showed enrichment in ROS (**Figure 5A**). In a volcano plot, CD45^+^EPCs showed high expression levels of genes related to ROS production (**Figure 5B**). These results were confirmed by flow cytometry (**Figure 5C**). Our findings are consistent with those of previous studies (12). To clarify the role of ROS in antigen-specific CD8^+^T cell tolerance mediated by CD45^+^EPCs *in vivo*, we treated CD8^+^T cells with a ROS inhibitor. The ROS inhibitor abrogated the tolerogenic effect of CD45^+^EPCs on CD8^+^T cells (**Figures 5D, E**). ROS interacts with NO to form the biologically active peroxynitrite (ONOO^-^) (14, 15). To evaluate the role of peroxynitrite in CD45^+^EPC-mediated CD8^+^T cell tolerance, mice were subjected to adoptive transfer of OT-1 CD8^+^T cells and CD45^+^EPCs and then treated with UA), which specifically neutralizes peroxynitrite. With UA treatment, CD45^+^EPC loaded with specific peptides did not reduce CD8^+^T cell proliferation or downregulate IFN-γ expression (**Figures 5F, G**). These findings indicate that UA treatment can reverse antigen-specific CD45^+^EPC-mediated CD8^+^ T-cell tolerance.

### 3.6 Role of CD45^+^EPC induced nitration of the TCR complex in CD8^+^T cell tolerance

**Figure 6:**
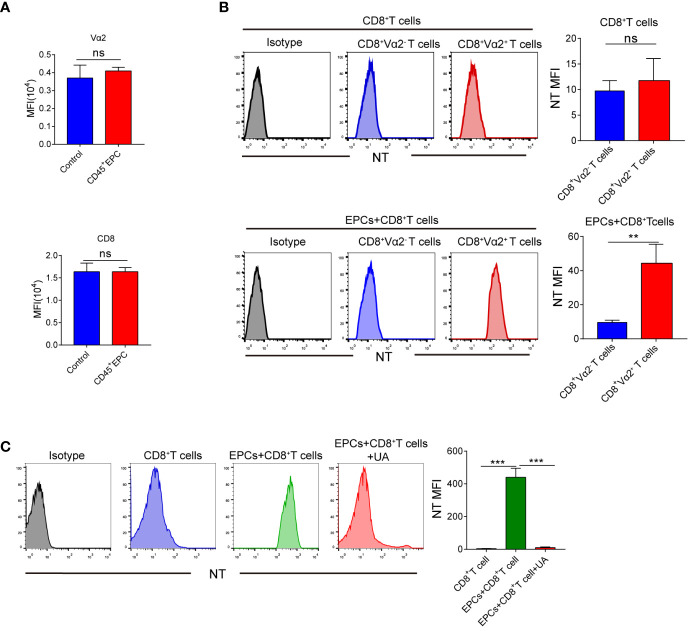
The role of CD45^+^EPC induced nitration of TCR complex in CD8^^+^^T cell tolerance. **(A)** Adoptive transfer and immunization was performed in **Figure 3** V_α2_ and CD8 MFI in CD8^+^T cells was detected by FACS. **(B)** Adoptive transfer and immunization was performed in **Figure 3**. Splenocytes were isolated and labeled with anti-CD8 and anti-V_α2_, and anti-NT antibodies. NT level in CD8^+^ V_α2^+^_ and CD8^+^ V_α2^-^_ cells was detected by FACS. **(C)** Splenocytes were cultured with CP or SP for 72h in presence of CD45^+^EPC. The levels of NT in CD8^+^T cells was evaluated by FACS after 72h. Two-way ANOVA was performed to assess significance, **p < 0.01, ***p < 0.001. ns, means no significance.

Several studies have demonstrated that peroxynitrite downregulates the expression of TCR and CD8 in T-cells (16–19). We speculated that this may be responsible for the antigen-specific CD8^+^T cell tolerance. However, TCR and CD8 expression did not differ significantly between CD8^+^T cells obtained from control mice and those obtained from mice that received CD45^+^EPCs (**Figure 6A**). Peroxynitrite can modify proteins by oxidizing or nitrating different amino acids. One of the major targets of peroxynitrite activity is tyrosine, which is converted to nitrotyrosine (NT) (20–22). The formation of NT increases the rigidity and disrupts the integrity of the pMHC-TCR complex (23–25). We investigated whether CD45^+^EPC could induce tyrosine nitration in the TCR and CD8 molecules in antigen-specific CD8^+^T cells. Splenocytes were obtained from mice after adoptive immunization with OT-1 CD8^+^T and CD45^+^EPCs. Donor peptide-specific CD8^+^TCR Vα2^+^ T and recipient CD8^+^TCR Vα2^-^T cells were gated, and the expression of NT on the cell surface was evaluated using an NT-specific antibody. CD8^+^TCR Vα2^+^ and CD8^+^TCR Vα2^-^ T cells from control immunized mice showed similar NT levels (**Figure 6B**). However, CD8^+^TCR Vα2^+^ T cells from CD45^+^EPC recipients had significantly higher NT levels (**Figure 6B**). Splenocytes were pre-activated with specific peptides for 72 h and then incubated for 48 h with CD45^+^EPCs isolated from tumor-bearing mice. CD45^+^EPC transfer significantly increased NT levels in OT-1 CD8^+^ T cells. Treatment of CD45^+^EPCs with the ROS inhibitor blocked the CD45^+^EPC-induced increase in NT levels (**Figure 6C**). These results suggest that CD45^+^EPCs induce nitration of the TCR complex.

### 3.7 UA treatment combined with CD8^+^T cell adoptive immunotherapy prevented CD45^+^EPC-induced tumor growth

**Figure 7:**
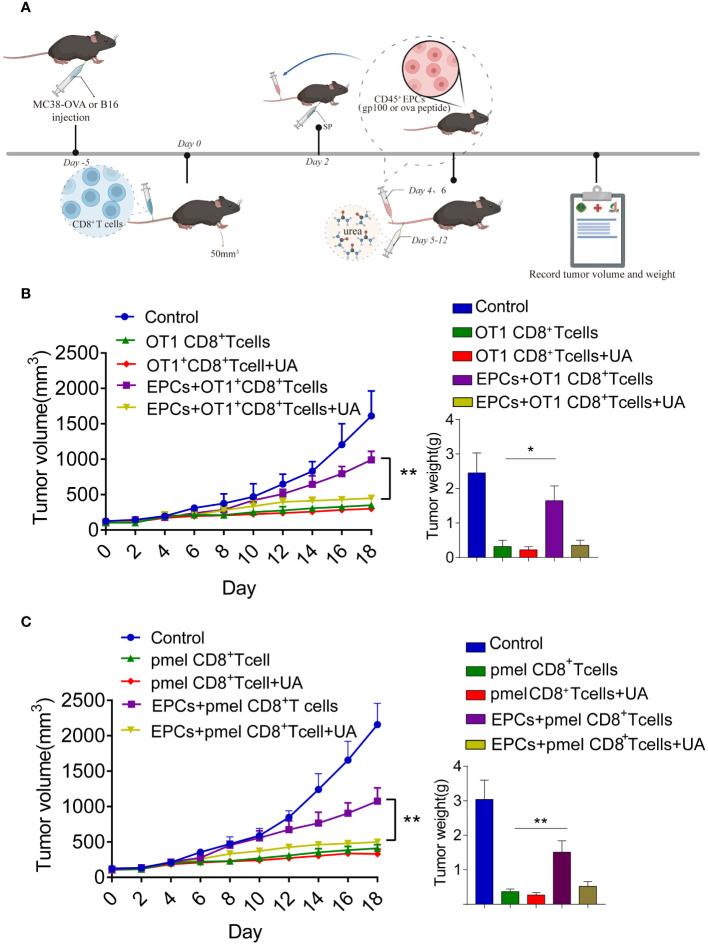
Combined UA with CD8^+^T cell adoptive immunotherapy treatment restrain CD45^+^EPCs induced-tumor growth. **(A)** Schematic illustration of treatment schedule. MC38-OVA or B16 tumor-bearing mice received SIINFEKL peptide or gp100 peptide on the indicated days. Treatment with UA was started three days after first immunization and was continued for two weeks. CD8^+^T cells and CD45^+^EPC were transferred into mice on the indicated days. Results represent the assessment of tumor growth differences at the last time point. **(B, C)** Tumor growth analysis in tumor-bearing mice (n = 6). Mean ± SEM is shown. *p<0.05, **p<0.01.

We further addressed the possibility that blocking peroxynitrite may improve the anti-tumor effects of CD8^+^T cell adoptive immunotherapy. MC38-OVA or B16F10 tumor cells were subcutaneously transplanted into C57BL/6 mice. On day 0, OT1 or PMEL CD8^+^T cells (2 × 10^6^) were intravenously transferred after tumor cell inoculation. Two days later, the mice were immunized with OVA or gp100. The mice were then intravenously injected with specific peptide-pulsed CD45^+^EPC isolated from the spleens of tumor-bearing mice (**Figure 7A**). UA treatment was started on day 4 and continued for 7 days. We found that the addition of UA potentiated the anti-tumor effects of antigen-specific CD8^+^T cells (**Figures 7B, C**). Additionally, we analyzed the status of the immune microenvironment after UA treatment. We discovered that UA treatment can reverse the tolerance of CD8^+^T cells mediated by CD45^+^EPCs and increase the expression levels of IFN-γ and TNF-α in CD8^+^T cells (**Supplementary Figures 2A, B**). UA treatment reduced the proportions of MDSCs and macrophages in tumor tissues and did not affect the proportions of Treg and CAF (**Supplementary Figures 2C–F**). Taken together, these results suggest that UA treatment not only reverses the tolerance of CD8^+^T cells mediated by CD45^+^EPCs but also improves the immune microenvironment and prevents tumor growth.

## 4 Discussion

T cells that are reactive against tumor antigens are found in patients with cancer and can selectively eliminate tumor cells (26). Over the last few decades, tumor antigens have been targets of immunotherapy, including adoptive T-cell therapies (27–29). Although the outcomes in hematological malignancies and melanoma are encouraging, successful treatments targeting self-antigens in solid tumors are limited (30, 31). Therefore, a deep understanding of the mechanisms underlying adoptive T-cell therapies in solid tumors and the factors contributing to their poor therapeutic effects is required. Previous studies have shown that APCs in the tumor microenvironment may be responsible for this phenomenon. Some studies have shown that dendritic cells pick up antigens from tumor cells and present them to Tregs within the tumor microenvironment or after migrating to tumor-draining lymph nodes (5, 32), resulting in immunosuppression. Similarly, MDSCs in the tumor microenvironment pick up and process soluble proteins, inducing antigen-specific tolerance in CD8^+^T cells (8). However, the mechanism of T-cell tolerance remains unclear. There is recent evidence that anemia is associated with a severe deficiency in CD8^+^ cell responses to pathogens in treatment-naïve mice with large tumors and identified CD45^+^EPCs as robust immunosuppressants (12). CD45^+^EPCs, induced by tumor growth-associated extramedullary hematopoiesis, accumulate in the spleen and become a major population. CD45^+^EPCs from patients with cancer and mice bearing large tumors contains many myeloid-expressing surface markers and gene signatures, apparently specifying an erythroid lineage (12). These findings suggest that CD45^+^EPCs and myeloid cells have similar biological characteristics. Accordingly, we hypothesized that CD45^+^EPCs mediate tumor-induced antigen-specific CD8^+^T cell tolerance.

To test this hypothesis, we first tested whether CD45^+^EPCs could pick up and process soluble proteins. We observed OVA/gp100 expression on the surface of CD45^+^EPCs after the addition of the Ova/gp100 peptide to the medium. We generated an *in vitro* model in which CD45^+^EPCs were pulsed with control or specific peptides from tumor-bearing mice and co-cultured with splenocytes that were pulsed with specific peptides. The results showed that CD45^+^EPCs loaded with specific peptides reduced CD8^+^T cell responses significantly. Furthermore, we constructed an *in vivo* model by adoptively transferring OT1 and PMEL CD8^+^T cells to tumor-free recipients. The results showed that tumor-derived CD45^+^EPC inhibited the response of CD8^+^T cells to specific antigens, suggesting that the inhibition of responsiveness is antigen-specific and MHC I-restricted. Taken together, these data indicate that CD45^+^EPCs induce antigen-specific CD8^+^T cell tolerance.

In tumor-free mice, CD45^+^EPC failed to inhibit immunosuppression, probably because CD45^+^EPCs from tumor-bearing mice produce higher levels of ROS than those of their control counterparts. However, the underlying molecular mechanisms remain unknown and need to be elucidated. The role of ROS in T cell defects has been demonstrated in several studies showing that ROS downregulates the expression of TCR and CD8 molecules in T cells. Therefore, we speculated that this may be responsible for antigen-specific CD8^+^T cell tolerance. We found that the expression levels of TCR and CD8 were similar in CD8^+^T cells obtained from control mice and those obtained from mice to which CD45^+^EPCs were transferred. ROS can also reduce the affinity of antigens to their specific TCRs, which can explain the specificity of the tolerance induced by CD45^+^EPC.

Our experiments demonstrated the critical role of ROS in CD45^+^EPC-mediated antigen-specific CD8^+^T cell tolerance. ROS can modify proteins either directly or in combination with NO, thereby contributing to peroxynitrite generation (33). In this study, the use of peroxynitrite scavengers eliminated CD45^+^EPC-mediated antigen-specific CD8^+^T cell tolerance. Tyrosine nitrification is a recently established marker of peroxynitrite activity. Some studies have revealed that tyrosine residues in TCR and CD8 molecules are susceptible to nitration (34). Moreover, nitration of these residues would lead to decreased flexibility and increased rigidity of the TCR domains, which might alter epitope-specific interactions between the TCR and pMHC substantially. Higher NT expression on the surface of antigen-specific CD8^+^ T cells from mice treated with CD45^+^EPC confirmed the antigenic specificity of the tolerance induced by contact with CD45^+^EPC.

From a therapeutic standpoint, our study outlines a strategy to effectively reduce the nitration of the TCR complex by UA and abolish the immunosuppressive effects of CD45^+^EPCs during CD8^+^T cell adoptive transfer, thereby tipping the balance toward effective anti-tumor immunity. These findings indicate that combining UA treatment with adoptive CD8^+^T cell or chimeric antigen receptor T-cell immunotherapy could be an effective therapeutic approach.

**Figure 8:**
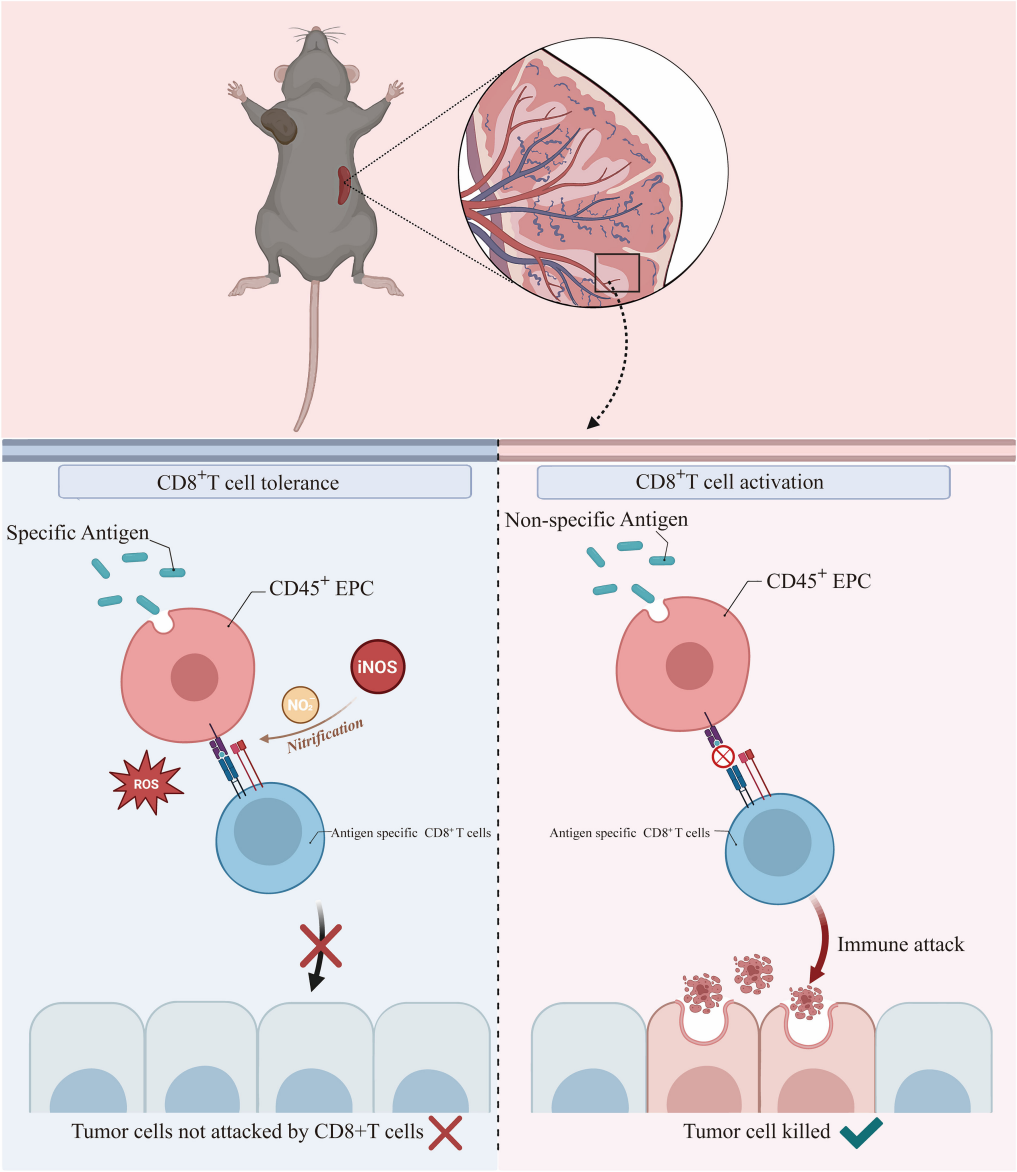
CD45^+^EPCs take up soluble proteins, present antigenic epitopes on their surface, and induce antigen-specific CD8^+^ T-cell tolerance by ROS, thereby suppressing anti-tumor immunity.

In summary, we demonstrated that CD45^+^EPCs take up soluble proteins, present antigenic epitopes on their surface, and induce antigen-specific CD8^+^ T-cell tolerance, thereby suppressing anti-tumor immunity. Mechanistically, ROS and peroxynitrite generated from CD45^+^EPCs induced the nitration of tyrosine in TCR/CD8 molecules, which induced antigen-specific non-responsiveness of CD8^+^T cells. Importantly, we also showed that abolishing CD45^+^EPC function with UA increases the anti-tumor efficacy of adoptive T-cell therapies (**Figure 8**).

## Data availability statement

The datasets presented in this study can be found in online repositories. The names of the repository/repositories and accession number(s) can be found in the article/**Supplementary Material**.

## Ethics statement

The animal study was approved by The Animal Ethical and Welfare Committee of Army Medical University. The study was conducted in accordance with the local legislation and institutional requirements.

## Author contributions

XF: Conceptualization, Data curation, Formal analysis, Investigation, Methodology, Project administration, Software, Writing – review & editing. HP: Conceptualization, Data curation, Formal analysis, Investigation, Methodology, Software, Validation, Visualization, Writing – review & editing. XW: Conceptualization, Data curation, Formal analysis, Investigation, Methodology, Software, Writing – review & editing. YS: Data curation, Formal analysis, Investigation, Methodology, Writing – review & editing. YD: Conceptualization, Investigation, Project administration, Resources, Supervision, Writing – review & editing. JZ: Writing – review & editing. JC: Conceptualization, Investigation, Methodology, Project administration, Resources, Supervision, Writing – review & editing. SH: Conceptualization, Funding acquisition, Investigation, Project administration, Resources, Supervision, Visualization, Writing – original draft, Writing – review & editing.
