# Supplementary material for: Tumor-associated CD8+T cell tolerance induced by erythroid progenitor cells
Source: Front Immunol. 2024 May 10;15:1381919. doi: 10.3389/fimmu.2024.1381919 (PMC11116624; doi:10.3389/fimmu.2024.1381919)
Supplement: Supplementary file 1 [file DataSheet_1.pdf]

Tumor-associated CD8$^+$T cell tolerance induced by erythroid progenitor cells

Xue Fan[3*], Han Peng[1,2*], Xuesong Wang [1,2*], Yixin Sun[1,2], Yan Dong[1,2], Jie Zhou[1,2], Jianfang Chen[1,2], Shuo Huang[1,2]

[1]Department of Oncology and Southwest Cancer Centre, Southwest Hospital, Third Military Medical University (Army Medical University), Chongqing 400038, China

[2]Radiation Treatment Centre, Southwest Hospital, Third Military Medical University (Army Medical University), Chongqing 400038, China

[3]Endocrinology/Osteoporosis Department, West China School of Public Health and West China Forth Hospital, Sichuan University, Chengdu 610041, China

Correspondence:
Jianfang Chen, Ph.D.
E-mail: cjf043@sina.com

Shuo Huang, Ph.D.
E-mail: 18180920941@163.com

Keywords: erythroid progenitor cell; CD45; peroxynitrite, immunotherapy

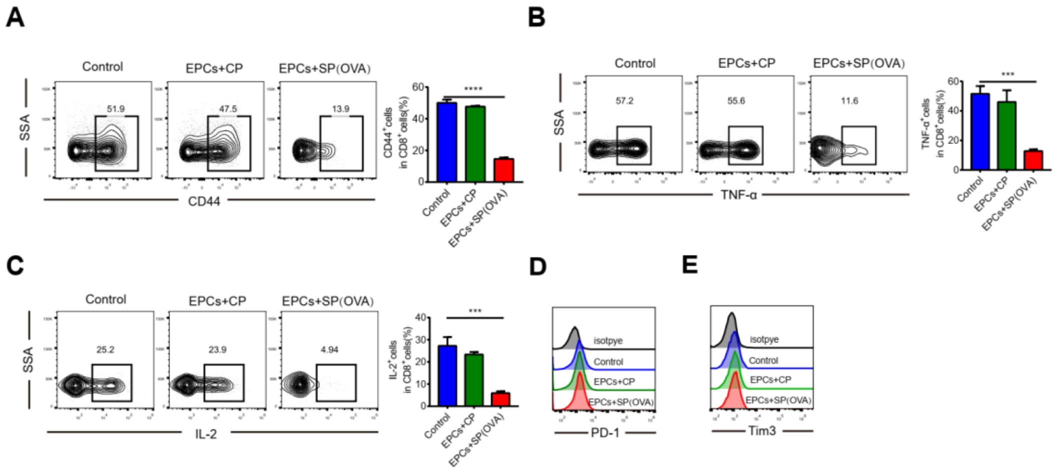

Supplementary Figure S1.

*(A-C) Percentage of CD44, TNF-α, IL-2 on CD45.1^{+}CD8^{+}T cells was analyzed by FACS(n=3). (D-E) PD1 and Tim3 expression on CD45.1^{+}CD8^{+}T cells was analyzed by FACS(n=3). One-way ANOVA was used to evaluate statistical significance(***p<0.001, ****p<0.0001).*

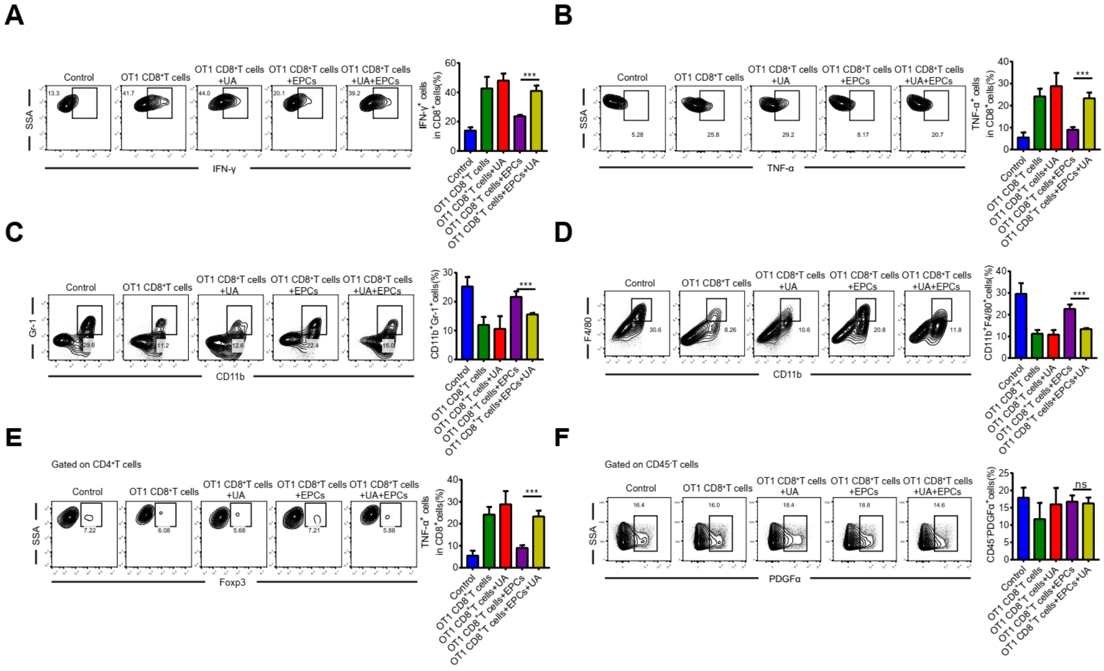

Supplementary Figure S2.

*(A-B) Percentage of IFN-γ, TNF-α on CD8⁺T cells in tumor tissuses after UA treatment was analyzed by FACS(n=6). (C-F) Percentage of MDSC(CD11b⁺Gr-1⁺), macrophage (CD11b⁺F4/80⁺), Treg (CD4⁺Foxp3⁺), CAF(CD45⁻PDGF⁺) in tumor tissuses after UA treatment was analyzed by FACS(n=6). Two-way ANOVA was performed to assess significance, ***p<0.001.*

Table S1

Antibodies for flow cytometry	catalog no.	Distributor
Anti-Ter119	116208	Biolegend
Anti-CD71	113812	Biolegend
Anti-CD8	162306	Biolegend
Anti-IFN-γ	505806	Biolegend
Anti-CD45	103128	Biolegend
Anti-Ki67	652410	Biolegend
Anti-OVA	O34781	Invitrogen
H-2Kb bound to SIINFEKL Antibody（25-D1.6）	141606	Biolegend
Anti-CD45.1	110726	Biolegend
CFSE	423801	Biolegend
anti-TCR Vα2	127813	Biolegend
Anti-NT	ab183390	Abcam
Fixable Viability Dye eFluor	65-0865-18	Ebioscience
Anti-PD-1	135215	Biolegend
Anti-Tim3	134003	Biolegend
Anti-CD44	156005	Biolegend
Anti-IL-2	503831	Biolegend
Anti-TNFα	506305	Biolegend
